# Imaging extracellular vesicles: current and emerging methods

**DOI:** 10.1186/s12929-018-0494-5

**Published:** 2018-12-24

**Authors:** Steven Ting-Yu Chuo, Jasper Che-Yung Chien, Charles Pin-Kuang Lai

**Affiliations:** 1 Institute of Atomic and Molecular Sciences, Academia Sinica, No. 1, Roosevelt Rd., Sec. 4, Taipei, 10617 Taiwan; 2 Chemical Biology and Molecular Biophysics Program, Taiwan International Graduate Program, Academia Sinica, Taipei, Taiwan; 3 Genome and Systems Biology Degree Program, National Taiwan University and Academia Sinica, Taipei, Taiwan

**Keywords:** Extracellular vesicles exosomes, Microvesicles, Imaging, Biodistribution, Fluorescence, Bioluminescence, MRI, SPECT, Dyes

## Abstract

Extracellular vesicles (EVs) are lipid bilayer-enclosed nanoparticles released by cells. They range from 30 nm to several micrometers in diameter, and ferry biological cargos such as proteins, lipids, RNAs and DNAs for local and distant intercellular communications. EVs have since been found to play a role in development, as well as in diseases including cancers. To elucidate the roles of EVs, researchers have established different methods to visualize and study their spatiotemporal properties. However, since EV are nanometer-sized, imaging them demands a full understanding of each labeling strategy to ensure accurate monitoring. This review covers current and emerging strategies for EV imaging for prospective studies.

## Background

Extracellular vesicles (EVs) are heterogeneous nanoparticles released by cells. They were once considered as cellular wastes until studies revealed that EV serves as a means of cell-to-cell communication by shuttling DNAs, RNAs, proteins and lipids to neighboring and distant sites [1, 2]. Since then, EVs have been actively investigated under (patho)physiological settings, as well as for therapeutic development. To aid in these studies, many methods have been developed to label and characterize the spatiotemporal property of EVs. As each imaging strategy carries its advantages and disadvantages, this review aims to cover current and emerging methods, thereby facilitating choice for EV imaging in prospective studies.

## Extracellular vesicles

**Fig. 1:**
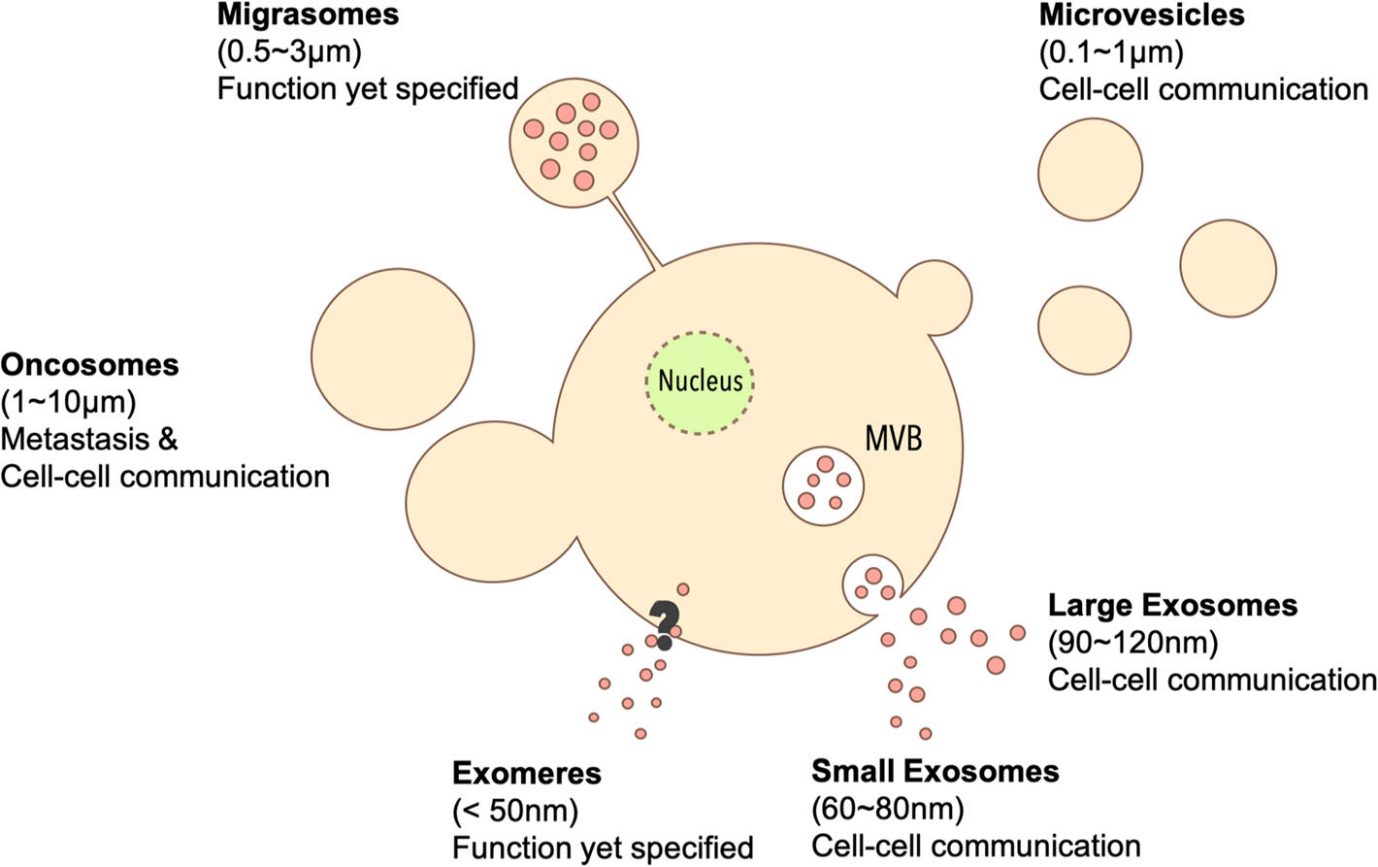
Schematic of different EV subpopulations. Different EV subtypes have different sizes and secretion pathways. Exosomes are generated from MVBs, and can carry protein and mRNAs cargo for cell-cell communication. Based on their sizes, exosomes can further characterized to small exosomes and large exosomes. Exomeres are nanoparticles with size smaller than 50 nm and carrying proteins involving metabolism; their biological role remains unknown. Microvesicles shed from the cell surface are generally larger than exosomes, and can also ferry cargos between cells. Oncosomes are larger EVs that were generated from cancer cells by budding or membrane scission, and can deliver cancer metastasis-related cargo to facilitate tumor cell invasion. Migrasomes are generated after cells migration with its function yet to be identified

Valadi et al. identified that EVs from human and mouse mast cell carry mRNAs and microRNAs (miRNAs) named “exosomal shuttle RNAs”, which could be delivered into recipient cells via EV uptake for translation [3]. Soon thereafter, Al-Nedawi et al. found EVs derived from gliomas could deliver an oncogenic form of EGFR (epidermal growth factor receptor), EGFRvIII [4], and further showed that EVs released by A431, A549 and DLD1 cancer cell lines could transfer EGFR to induce angiogenesis in human umbilical vein endothelial cells (HUVECs) [5]. Moreover, Ratajczak et al. discovered that EVs from embryonic stem cell (ES) could deliver mRNAs related to pluripotent transcription factors and Wnt-3 protein to murine hematopoietic progenitor cells (HPC) to enhance survival and expansion [6]. Since EVs could transport bioactive cargos between cells, EVs are recognized as important carriers to modulate phenotype and function of EV recipient cells [7]. While there are different EV subtypes based to their size, biogenesis and shape (Fig. 1), the collective term “EVs” is used in the current review unless otherwise specified.

*Exosomes* are nanosized vesicles (30–100 nm) generated by the release of intraluminal vesicles following the fusion of multivesicular bodies (MVBs) with the plasma membrane. Cells first generate early endosomes by endocytosis [8–10]. During their maturation to late endosomes, some endosomes shed intraluminal vesicles (ILVs) within itself to become MVBs, which then migrate to the cell membrane [11]. Once fused with the plasma membrane, the MVBs release the vesicles within to the extracellular milieu as exosomes [12]. Since exosomes are generated from the MVBs, exosomes contain biomarkers such as Alix and tumor susceptibility gene 101 (Tsg101) which relate to ILV formation in endosomal sorting complex required for transport (ESCRT) [13]. Exosomes from dendritic cells, HeLa cells, human embryonic kidney cells 293 T (HEK293T) and retinal pigmented epithelial cells (RPE-1) were discovered to have tetraspanins like CD9, CD81 and CD63 which relate to endosomal vesicle trafficking [14, 15]. Therefore, these tetraspanins are also considered as common exosomal markers.

Zhang et al. identified two kind of subpopulations of exosomes: *large exosomes* (Exo-L, 90–120 nm) and *small exosomes* (Exo-S, 60–80 nm) by asymmetric flow field-flow fraction (AF4) [16]. Using transmission electron microscopy (TEM) and mass spectrometry (MS) analysis, Exo-S/L were confirmed as encapsulated particles [15]. While both Exo-S/L exhibited similar biomarkers as exosomes including tetraspanins (CD9, CD63, CD81), Exo-S contained canonical exosomal proteins relating to ILVs, phagocytic vesicles, MVB and vacuoles like flotillin 1, flotillin 2, tweety family member 3, tetraspanin 14 and ESCRT-I subunit VPS37B. By contrast, Exo-L carried non-canonical proteins associated with membrane budding, late-endosome and trans-Golgi network such as annexin A1/A4/A5, charged multivesicular body protein 1A/2A/4B/5, vacuolar protein sorting 4 homologue B, heat shock protein family (Hsp40) member A1 and myosin IC. Furthermore, Zhang et al. identified a smaller, non-membranous nanoparticle named “*exomere*” (< 50 nm), which lacks the lipid bilayer of other EV subtypes [15]. Exomeres are enriched with proteins involved in metabolism including glycolysis and mTORC1 metabolic pathway [15], and its biological role remains to be elucidated in upcoming investigations.

*Microvesicles* (100–1000 nm) are shed from the surface of cells which are generally larger than exosomes. The outward budding is related to the interaction of TSG101 with arrestin domain-containing protein 1 (ARRDC1). After binding with ARRDC1, TSG101 relocates from endosomes to the plasma membrane and facilitate MV release through Gag-mediated budding [17, 18]. MVs share some of the biomarkers with exosomes like CD63 [19], and both MVs and exosomes are known to transport bioactive cargos between cells [6].

*Oncosomes or large oncosomes* are large EVs released by cancer cells (1000–10,000 nm). They could be released like microvesicles by vesicle budding and membrane scission [20, 21] Oncosomes are frequently found in highly aggressive cancer cells as non-apoptotic plasma membrane blebs during amoeboid mode of cancer invasion [22]. Wolf et al. discovered that amoeboid-like tumor cells continuously expand and retract oncosomes around cell surface when tumor cells go through 3D collagen matrix [22]. Clancy et al. found the release of oncosomes from amoeboid-like invasive tumor cell, which is facilitated by soluble N-ethylmaleimide-sensitive-factor attachment protein receptor (SNARE) protein and vesicle-associated membrane protein (VAMP) with cargo delivery of membrane-type 1 matrix metalloprotease (MT1-MMP) [21, 23, 24]. Since MT1-MMP is a facilitator of tumor cell invasion and extracellular matrix (ECM) proteolysis [25, 26], oncosomes are suggested to play an important role in tumor cell invasion.

*Migrasomes* (up to 3000 nm) are oval shaped microvesicles containing small vesicles formed during cell migration. Liang et al. discovered that cells secrete migrasomes from tips of their retraction fibers, which the authors described as pomegranate-like structures (PLS) [27]. PLS were found to express tetraspanin-4 (TSPAN4) as a PLS marker [27]. With time-lapse fluorescence imaging of TSPAN4-green fluorescent protein (GFP)-expressing normal rat kidney (NRK) cells, the authors found migrasome release was migration-dependent [27]. Yet, migrasome function remains to be elucidated.

Although EV subtypes have different routes of biogenesis, biomarkers and sizes, their respective biological roles remain to be fully characterized. With the recent advances in EV labeling and imaging technologies, a more comprehensive understanding on the properties of EV subtypes may be made possible.

## EV imaging

**Fig. 2:**
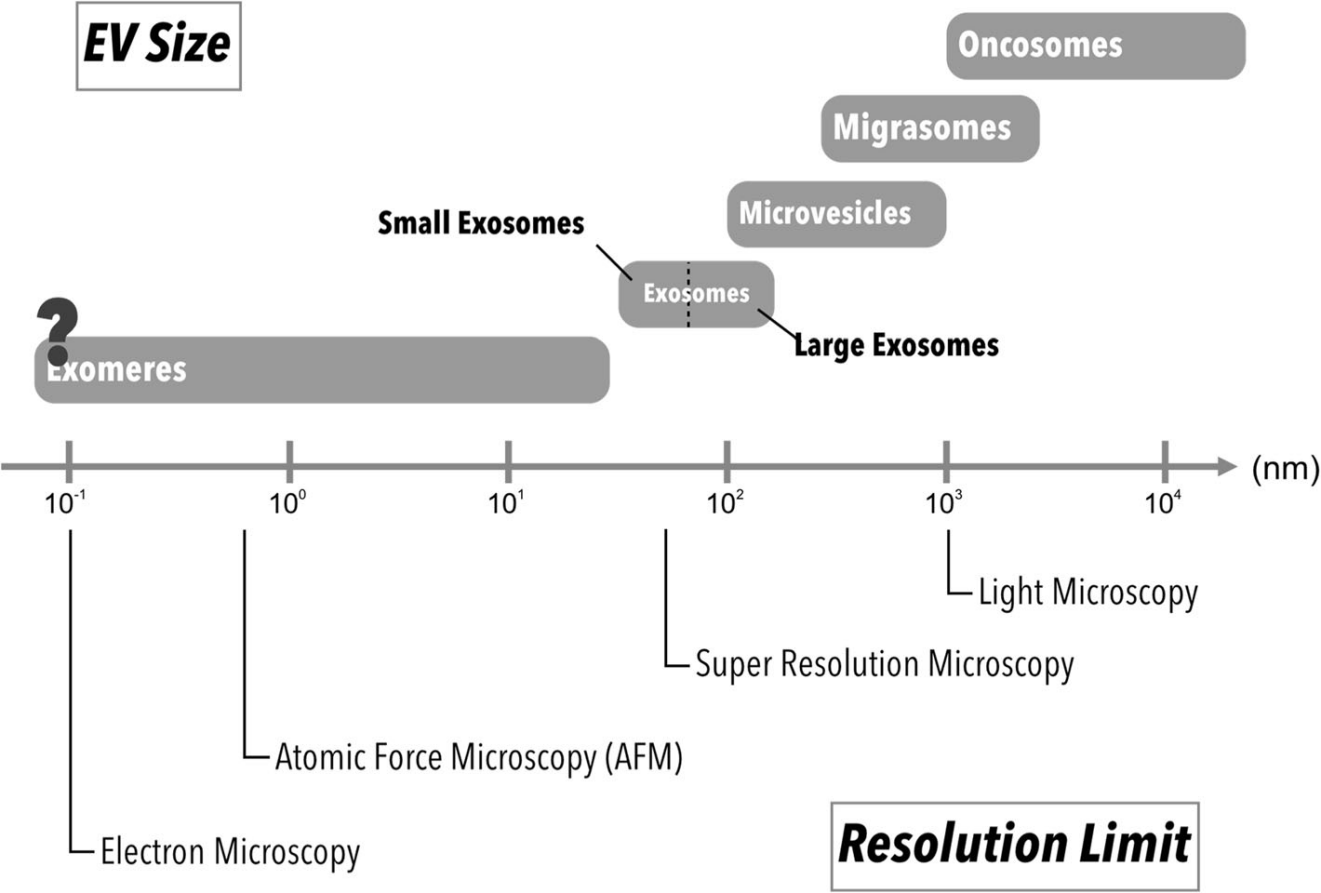
Different microscopic resolution limits and sizes of EV subpopulations. Each imaging method has its resolution limit. Different strategy can be applied for EV imaging based on EV subtypes and target(s) of interest (e.g. cells, tissues, organs)

**Fig. 3:**
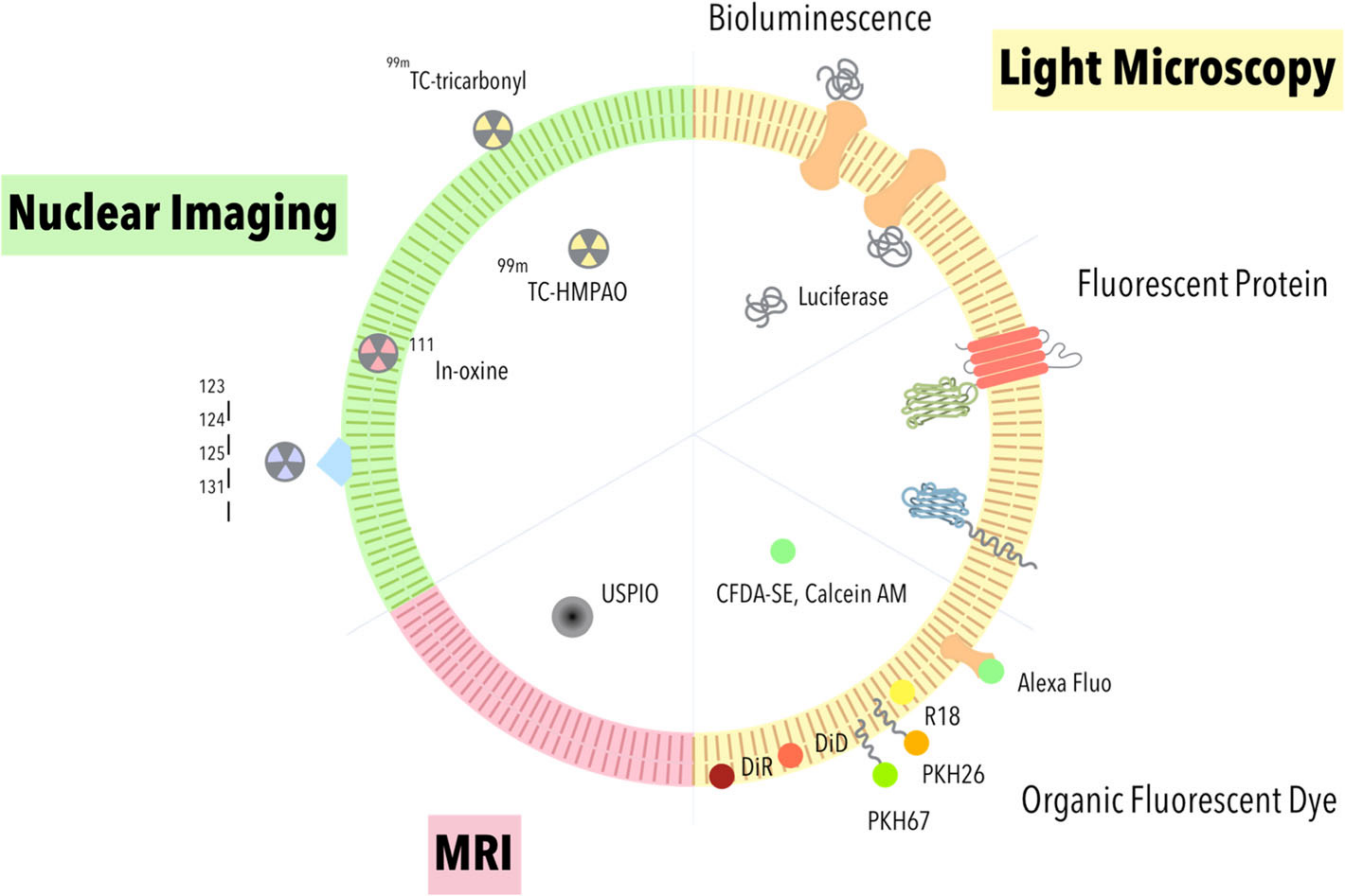
Strategies for EV labeling and imaging. Labeling EV with fluorescent dye or fluorescent protein can be imaged by fluorescent microscopy. EVs expressing bioluminescence proteins can be imaged by ultra-sensitive CCD. EVs incorporated with USPIO can be used for MRI imaging. EVs label with isotopes can be used for nuclear imaging. CFDA-SE: carboxyfluorescein diacetate succinimidyl ester; calcein AM: calcein acetoxymethyl; USPIO: ultra-small super paramagnetic iron oxide; 99mTc-HMPAO: 99mTc-hexamethylpropylene-amineoxime; CCD: charge-coupled device

EV imaging plays an important role in revealing spatiotemporal property of EVs to further our understandings in the molecular biology, as well as therapeutic potential of EVs. In vitro EV imaging helps researchers to understand the physical property of EVs such as the mechanism of EV release [28] and uptake [1, 29], or biomarkers expressed on the EV surface [30, 31]. In vivo EV imaging aids in unveiling the biodistribution of EVs, which can be used to characterize pharmacokinetic property of EVs as a drug and/or theranostics vehicle. However, imaging and tracking EVs can be challenging due to their small sizes, often requiring labeling prior to their subsequent visualization (Fig. 2). Many imaging tools and labeling methods have since been developed to assist researchers in monitoring EVs both in vitro and in vivo (Fig. 3). In this review, we will focus on the advantages and disadvantages of commonly used methods for EV visualization for basic and preclinical studies.

## EV imaging with Electron microscopy

Electron microscopy has been considered as a standard imaging method for observing nanosized samples, including EVs [32–34]. Since electron microscopy typically has a resolution around 0.5 nm which is smaller than exosomes, it may provide detailed structural information of EVs. It is important to note that electron microscopy cannot image EVs in their native state because the samples need to be fixed and processed prior to imaging. Here we will discuss the common electron microscopy methods used for EV imaging:

## Transmission Electron microscopy

Transmission electron microscopy (TEM) is the most common type of electron microscopies for EV imaging, such as exosomes [35], microvesicles [36], oncosomes [37] and migrasomes [27]. The samples prepared for TEM imaging are first fixed and later dehydrated. Following dehydration, the samples need to be embedded, sliced into nanometered thin sections, and mounted on a carbon coating grid for imaging. TEM uses electron beams to illuminate through prepared specimens, and the electron can either transmit or be diffracted by the specimens. A fluorescent screen or charge-couple device (CCD) will collect the transmitted electron for bright-field images, which is normally used for structure verification. Meanwhile, scattered electrons are collected to generate dark-field images, revealing the structure with a higher contrast. Notably, EVs observed by TEM often appear as cup shaped as a result of dehydration during sample preparation [38] but can effectively reveal inner structure of EVs.

Using immunogold-labeling, TEM can further reveal EV proteins. Dickens et al. used correlative light-electron microscopy (CLEM) to visualize EVs released from GFP-expressing astrocytes, thereby demonstrating that the labeled EVs can be taken up by brain microvascular endothelial cells, the lung, liver and spleen, and subsequently induce leukocytes migration to brain lesion tissues [39]. The immunogold-labeled method can also be used to quantitate cancer-associated marker from plasma EVs [40], as well as to study disease mechanism involving EVs. For instance, Szempruch et al. recently found EVs secreted from a parasite, *Trypanosoma brucei*, causes host erythrocyte remodeling and subsequent anemia [41].

### Scanning Electron microscopy

Scanning electron microscopy (SEM) uses electron beam to scan the surface of specimen to generate topography information. For SEM, samples are first chemically or cryogenically fixed followed by dehydration. The immobilized samples are then sputter-coated with a thin layer of conductive material such as gold or carbon for imaging. While some reports suggest EVs under SEM as round shaped [42–44], others report them as saucer shaped [45]. The latter observation may reflect EV collapse as a result of the dehydration process during sample preparation [45].

Although SEM sample preparation is relatively simple when compared to that of TEM, which requires samples to be embedded and sectioned, several caveats need to considered. During sample preparation for SEM, a thin conductive layer around 2 to 10 nm is sputtered on the surface of sample to avoid accumulation of electron and to increase secondary electron generation. This thin layer of gold does not usually affect the imaging result. However, due to the small size of EVs, the thin layer of gold may affect the surface structure of EVs. A low-voltage SEM can avoid accumulation of charge and reduce radiation damage to the samples, thus bypassing the sputter coating process [44]. Chernyshev et al. also reported that “coffee ring effect” may occur as a result of the capillary flow during sample dehydration, thus creating bias in the result of EV size and amount [46]. To prevent such bias, the entire surface of specimen must be imaged and investigated [46].

### Cryo-electron microscopy

In cryo-electron microscopy (cryo-EM), samples will be fixed by cryo-immobilization where waters are vitrified instead of ice crystal formation in the sample by liquid ethane cooling. Cryo-immobilizing allows samples to be preserved in their native hydrated state, thus avoiding artifacts commonly caused by conventional fixation method such as cup shaped EVs [46, 47]. Combined with immunogold labeling, cryo-TEM can image EVs containing proteins and track EV uptake by recipient cells [48], as well as distinguishing EV subgroups by their size [49, 50]. Under cryo-EM, the specimens are imaged under extremely low temperature (below − 175 °C) as EVs are maintained in its original spherical shape [51]. Therefore the average size of EVs will appear to be bigger when compared to other EM methods [46]. After cryo-immobilization, samples can also undergo freezing substitution with fixing and embedding reagents for the specimens to be imaged under traditional TEM in room temperature. Since cryo-EM yields superior sample quality and morphology preservation over traditional EM methods [47], it is increasingly being applied to study EVs.

### EV imaging with atomic force microscopy

Atomic force microscopy (AFM) uses a probe often made by silicon or silicon nitride to scan through the surface of specimens. When the probe contacts with the surface of specimens, the probe position changes and is measured by a laser beam. By recording the probe position during the scan, AFM generates topographic images of the samples. AFM has a resolution limit around 1 nm [52], which allows quantification and imaging of most EVs [53, 54]. In air-mode, the sample preparation for EVs imaging only requires EVs immobilized on freshly cleaved mica for subsequent scanning with a probe. In liquid-mode, EV samples can be measured directly and will result in detection of larger sized EVs than that of the air-mode because EVs remain hydrated and maintain their morphology [55]. The mica can also be coated with antibodies so that EVs with specific antigen can be captured for imaging [54]. The imaging mode can be classified into contact and tapping mode. In contact mode, a probe scans across the surface of a sample, thus can damage both the probe and the sample. Whereas in tapping mode, probe oscillates across the sample surface and only touches the sample at the lowest position of oscillation. The oscillation reduces the contact time between the sample and the probe, thereby protecting the sample structure. When combining silicon probes with antibodies, AFM can further be used to quantify and image EVs with specific protein on its surface at single EV resolution [56].

## Tracking EV by optical microscopy

Bioluminescence imaging (BLI) and fluorescence imaging (FLI) are two major methods used in detecting EVs within the visible light spectrum (390–700 nm). Bioluminescence is a type of chemiluminescence produced from the oxidation of substrates by their respective luciferases. The bioluminescent signal requires ultra-sensitive CCD camera for detection [57]. An advantage of BLI lies in its high signal-to-noise ratio (SNR) since the signals are generated without any light source. FLI uses fluorescent proteins or organic dyes to emit signals under excitation with an external light source. When compared to BLI, FLI signal could be more easily detected by a CCD camera. Both BLI and FLI can be applied for real-time observation of EVs [58, 59].

### Bioluminescence EV labeling

BLI labeling of EVs are protein-based labeling. The EV-reporter luciferases are typically expressed in cells through plasmid transfection or lentivirus transduction, and their EVs can then be imaged via BLI.

Takahashi et al. demonstrated that *Gaussia* luciferase (Gluc) fused between a secretion signal peptide and C1C2 domain of lactadherin could be labeled onto EV membrane [59]. B16-BL6 murine melanoma cells were transfected with Gluc-lactadherin plasmid for 24 h and EVs were collected by differential ultracentrifuge (UC). After intravenous (IV) bolus injection of the labeled EVs, the signal showed that the EVs were quickly distributed to different organs within five hours [59].

We combined Gluc, biotin acceptor protein and the transmembrane domain of platelet-derived growth factor receptor (PDGFR) to create a multimodal EV imaging reporter (GlucB) [60]. Human embryonic kidney 293 T cells were stably transduced with a lentiviral vector containing GlucB for subsequent EV collection by differential UC. A bolus IV-administration of the labeled EVs into athymic nude mice followed by in vivo imaging system (IVIS) and fluorescence-mediated tomography demonstrated that EVs are mostly processed by the liver and lung over a period of six hours in two phases: a distribution phase where the EVs are quickly distributed to the different organs, and followed by an elimination phase where the EVs are processed by the organs [60].

Gangadaran et al. used *Renilla* luciferase (Rluc) as a BLI reporter for EV imaging. Lentivirus encoding Rluc was transduced into human anaplastic thyroid cancer (CAL-62 cells) and human breast cancer (MDA-MB-231) cells for EV isolation [61]. The labeled EVs showed biodistribution of EV-CAL-62/Rluc at the lung followed by the liver, spleen and kidney. On the other hand, EV-MDA-231/Rluc showed a strong signal at the liver followed by the lung, spleen and kidney [61].

Gluc and Rluc hence can serve as powerful reporters for in vivo EV biodistribution and imaging analyses. However, the toxicity of the substrates (e.g. coelenterazine) and half-life of bioluminescence should also be taken into consideration for BLI-based, real-time EV tracking [62–64].

### Fluorescence EV labeling

Fluorescent protein- and organic dye-based labeling are used to enable FLI EV imaging with excellent spatial resolution under optical microscopy and IVIS.

#### Recombinant protein labeling

Fluorescent proteins like GFP and RFP are fused with EV proteins as reporters for EV imaging. Mittelbrunn et al. first fused CD63 with GFP to analyze cellular uptake of EVs [31]. They generated stable CD63-GFP-expressing Raji B cell and J77 T cells to collect fluorescently labeled EVs. After 16 h EV treatment with CD63-GFP EVs to wildtype J77 T cells or Raji B cells, fluorescent signal was detected on recipient cell surface, indicating that EVs were attaching onto the cell membrane [31]. Suetsugu et al. used a similar strategy and showed that breast cancer cells secrete EVs to lung and induced cancer cell migration [65]. Another study used RFP tagged CD63 to image EV transfer between triple-negative breast cancer (TNBC) and macrophages RAW264.7 [66]. The communication between TNBC and macrophage through EVs causes M2-macrophage polarization and enhances tumor growth and axillary lymph node metastasis in orthotopic tumor models [66]. Yet, labeling EVs with specific EV proteins may limit the tracking to only a few subtypes of EVs expressing the respective markers.

To create a general labeling strategy of EVs with fluorescent proteins, we fused a palmitoylation signal to enhanced green fluorescence protein (PalmGFP) and tandem dimer Tomato (PalmtdTomato) to label the inner membrane leaflet of cells and EVs [67]. By using live-cell confocal microscopy, glioblastoma cells (GBM) and 293 T cells expressing the reporters showed multi-directional EV exchange [67]. Moreover, the reporters enabled in vivo observation of endogenously released EVs of implanted EL4 thymoma in C57BL/6 mice by multiphoton intravital microscopy (MP-IVM) [58].

Although fluorescent protein labeling methods could serve as versatile EV reporters, the fluorescence intensity depends on protein expression level, the efficiency of EV membrane domain labeling, and the strength of excitation light source. The expression of fluorescent proteins on EV membrane may also affect EV cargo content and uptake due to sterical hindrance, which require further investigations and consideration prior to their use.

#### Organic fluorescent dyes

There are many organic fluorescent dyes used for EV labeling. Most of the dyes were initially used to label cell membrane for imaging of cells. The organic dyes generally combine fluorophores with different functional groups to label the lipid bilayer or proteins of interest on EVs.

DiR and DiD are lipophilic dyes and exhibit a strong fluorescent signal when incorporated into the cytosol [68]. Wiklander et al. used DiR to study EVs by labeling conditioned media from different cell types followed by differential UC, and reported different EV biodistribution pattern based on cell and routes of administration in mice via IVIS [69]. Grange et al. also demonstrated that distributions of mesenchymal stem cell (MSC)-derived EVs were detectable through DiD labeling 24 h post-injection in mice [70]. PKH67 and PKH26 are also fluorophores with lipophilic carbocyanine. These dyes use aliphatic tails to anchor into lipid bilayer for fluorescence imaging [71, 72]. The lipophilic PKH dyes have also been used to label EVs to study in vivo properties [73, 74].

Octadecyl rhodamine B chloride (R18) is a lipid labeling dye that incorporates into lipid bilayer with its alkyl tails [75]. When first incorporated into the plasma membrane in quenched form, the intensity of R18 fluorescence signal increases as the labeled membrane fuses with unlabeled membrane to dequench R18 [76]. The percentage of dequenching can hence report EV fusion with cells [76]. Tian et al. used R18 to study fusogenic properties of EVs in PC12 cells and found fusion events in 24 h following EV treatment. Montecalvo et al. also used the same dye to detect bone marrow dendritic cell (BMDC) derived EVs fusing with BMDC within eight minutes following treatment [76].

Other water-soluble fluorophore combined with different functional groups are also applied to label EVs. Alexa Fluor NHS, a fluorescent dye bound with N-hydroxy succinimidyl (NHS) ester, can form covalent bond with amine groups in proteins [77]. Proteins present on EV lipid membrane can be labeled by Alexa Fluor NHS ester and detected by fluorescence imaging [78]. Kooijmans et al. used Alexa Fluor 488 to detect uptake of red blood cell-derived EVs by human epidermoid carcinoma cells, and found EVs decorated with EGFR sensitive nanobodies (EGa1-C1C2) could increase its uptake by flow cytometry analysis [79]. We showed that biotin acceptor protein in GlucB reporter can be further tagged with streptavidin-conjugated Alexa680 to enable fluorescence-mediated tomography (FMT) in mice to study biodistribution of 293 T-derived EVs [60].

Carboxyfluorescein diacetate succinimidyl ester (CFDA-SE; Ex/Em 492/517) is cell permeable and binds to intracellular amine group as it is retained in cells following removal of the acetate groups by intracellular esterases [80]. Escrevente et al. used CFDA-SE to observe energy-dependent endocytosis of EV uptake by SKOV3 cell (ovarian cancer cells) via flow cytometry [81]. CellTracker deep red (CTDR) has a similar function as CFDA-SE but with red light excitation (max. 630 nm) and far-red emission (max. 650 nm). When studying the cell uptake mechanism, CTDR labeled 239 T-derived EVs can be detected in green fluorescent dye labeled cells by fluorescence microscopic and flow cytometry analyses [82]. Calcein acetoxymethyl (AM) consists of fluorescent calcein combined with acetoxymethyl group. Calcein AM first penetrates into EVs with AM and is digested by cytosolic esterase to leave calcein as water-soluble fluorophore for FLI. Mantel et al. found calcein-AM could release calcein into RBC-derived EVs for observation using fluorescence microscopy and flow cytometry [83].

Fluorescent dyes can provide stable and strong signal for EV imaging. However, popular EV labeling dyes like PKH dyes has been reported to have an in vivo half-life ranging from 5 to > 100 days [84–86], and dialkylcarbocyanine dyes such as DiR could last for 4 weeks [87]. The persistence of the dyes may mislead the in vivo distribution in longitudinal studies of EVs where the dyes outlast EVs from degradation. Moreover, aggregation and micelle formation of lipophilic dyes may yield false signal of EVs [67]. Nevertheless, the dyes may be useful serving as a tracer to show where the EVs have traversed.

## Clinical imaging tools for EVs imaging

As researchers increasingly focus on EVs as an endogenous therapeutic delivery vehicle for clinical applications, one must be able to track and understand the pharmacokinetics of EVs. Two widely used clinical imaging tools are single photon emission computed tomography (SPECT) and positron emission tomography (PET). SPECT creates images by measuring gamma rays generated from gamma-emitting radioisotopes. By contrast, PET detects gamma ray pairs in an opposite direction when indirectly generated by positron-emitting radionuclide as it undergoes annihilation event with electrons in tissues. A major advantage of radioactive probes lies in its superior tissue penetration depth over visible light reporters.

Hwang et al. used lipophilic ^99m^Tc-hexamethylpropylene-amineoxime (^99m^Tc-HMPAO) to label EVs where the contrast agent was first trapped inside the macrophages as glutathione converts ^99m^Tc-HMPAO to hydrophilic form, subsequently generating ^99m^Tc-HMPAO exosome-mimetic nanovesicles through extrusion [88]. The ^99m^Tc-HMPAO-labeled nanovesicles showed a similar morphology and biodistribution pattern in mice as that of natural EVs, which are similarly labeled and collected by differential UC [88]. Similar method using ^99m^Tc-tricarbonyl complex, which binds to histidine [89], cysteine and methionine on surface proteins of EVs, enabled SPECT/CT imaging of erythrocyte-derived exosomes [90]. Another radiolabeling method involves the use of indium-111-oxine, which incorporates into exosome membrane with the lipophilic property of oxine [91]. Morishita et al. also developed an outer-membrane-labeling method using a fusion protein of streptavidin and lactadherin, a protein known to locate to the outer surface of exosomes. The labeled EVs are then treated with (3-^125^I-iodobenzoyl) norbiotinamide (^125^I-IBB) to label EVs via the biotin-streptavidin interaction [92]. Other common radioactive iodine, such as ^124^I, which is a common probe of PET [93], or ^131^I, which can kill and image cancer cells simultaneously [94], may also be used to radiolabel EVs in the future.

Magnetic resonance imaging (MRI) is another major molecular imaging technology used for clinical diagnosis. MRI contrast agent such as superparamagnetic iron oxide, which can reduce T2 signal in tissue, are commonly applied to improve signal-to-noise and lesion detectability [95]. In fact, Hood et al. used electroporation to load 5 nm superparamagnetic iron oxide nanoparticle into EVs, and demonstrated that the labeling did not affect their size and biodistribution in lymph nodes when compared to that of Dil labeled EVs in mice [96, 97]. Of note, since the electroporation method was also being used for cell or liposome fusion [98, 99], it may also cause EV fusion and affect their morphology. To avoid this caveat, an alternative EV labeling method employs cellular endocytosis of contrast agent. Hu et al. used ultra-small superparamagnetic iron oxide nanoparticles (USPIO, 4–6 nm) to label adipose stem cell through pinocytosis [100]. The internalized USPIO were then accumulated in MVB and released as USPIO-labeled EVs [101]. This method thus avoids EV fusion caused by electroporation, and tracks EVs release from implanted USPIO-labeled cells.

Although using SPECT, PET, MRI imaging system may provide good imaging depth, it is important to note that these labeling compounds have longer half-life than EVs and thus may generate signal even after EVs are degraded [88, 96].

## Conclusions

EVs imaging plays a pivotal role in studying biological phenomena such as cancers [102] and neuronal diseases [103]. As researchers utilize various reporters to monitor EVs, it is paramount to consider each reporter’s property in relation to that of EVs. It is also important to mitigate false positive EV signal from EV labeling, as well as to characterize true spatiotemporal property of EV but not the imaging agents. With progressively discovered information on EV biology and composition, new imaging methods may be developed to enable accurate, long-term imaging of EVs for preclinical and clinical settings.

## Abbreviations

AF4: asymmetric flow field-flow fraction
AFM: atomic force microscopy
AM: acetoxymethyl
ARRDC1: arrestin domain-containing protein 1
BLI: bioluminescence imaging
BMDC: bone marrow dendritic cell
CCD: charge-couple device
CFDA-SE: carboxyfluorescein diacetate succinimidyl ester
Cryo-EM: cryo-electron microscopy
CTDR: CellTracker deep red
CTZ: coelenterazine
ECM: extracellular matrix
EGFP: enhanced green fluorescence protein
EGFR: epidermal growth factor receptor
ES: embryonic stem cell
ESCRT: Endosomal sorting complex required for transport
EVs: Extracellular vesicles
Exo-L: large exosome
Exo-S: Small exosome
FLI: Fluorescence imaging
FMT: Fluorescence-mediated tomography
GFP: green fluorescent protein
Gluc: *Gaussia* luciferase
HEK293T: human embryonic kidney cells 293 T
HPC: hematopoietic progenitor cell
HUVAC: human umbilical vein endothelial cell
ILVs: Intraluminal vesicles
IVIS: in vivo imaging system
MP-IVM: multiphoton intravital microscopy
MRI: magnetic resonance imaging
MS: mass spectrometer
MSCs: mesenchymal stromal cells
MT1-MMP: cargo delivery of membrane-type 1 matrix metalloprotease
MVB: multivesicular body
NHS: N-hydroxy succinimidyl
NRK: normal rat kidney
OVA: chicken egg ovalbumin
Palm: Palmitoylation
PET: positron emission tomography
PLS: pomegranate-like structures
RFP: red fluorescent protein
Rluc: *Renilla* luciferase
RPE-1: retinal pigmented epithelial cells
SEM: scanning electron microscopy
SNARE: soluble N-ethylmaleimide-sensitive-factor attachment protein receptor
SNR: signal-to-noise ratio
SPECT: single photon emission computed tomography
tdTomato: tandem dimer Tomato
TEM: transmission electron microscopy
TNBC: triple-negative breast cancer
Tsg101: tumor susceptibility gene 101
TSPAN4: tetraspanin-4
UC: ultracentrifuge
USPIO: ultrasmall superparamagnetic iron oxide nanoparticles
VAMP: vesicle-associated membrane protein
^125^I-IBB: (3-^125^I-iodobenzoyl) norbiotinamide
^99m^Tc-HMPAO: ^99m^Tc- hexamethylpropylene-amineoxime
